# 肺鳞状细胞癌的遗传学特点及靶向治疗进展

**DOI:** 10.3779/j.issn.1009-3419.2015.03.08

**Published:** 2015-03-20

**Authors:** 晶 姜, 秀威 孙, 锦红 朱, 建群 马, 瑾薇 栾, 珊珊 刘

**Affiliations:** 150000 哈尔滨，哈尔滨医科大学附属肿瘤医院门诊化疗科 Department of Oncology, Harbin Medical University Cancer Hospital , Harbin 150000, China

肺癌是全世界癌症死亡的首要原因，在我国，肺癌也是发病率和死亡率最高的恶性肿瘤。肺癌按组织病理学主要分成两大类：小细胞肺癌（small cell lung cancer, SCLC）和非小细胞肺癌（non-small cell lung cancer, NSCLC）。NSCLC主要又分成鳞状细胞癌（squamous cell carcinoma, SQCC）、腺状细胞癌（adenocarcinoma, ADC）和大细胞癌（large cell carcinoma, LCC）。其中SQCC和ADC是NSCLC的两大主要组织病理学类型。近年来，在NSCLC中，尤其在ADC中，国内外学者陆续报道了表皮生长因子受体（epidermal growth factor receptor, *EGFR*）、*KRAS*等基因的突变以及针对这些驱动基因的靶向药物治疗给ADC患者带来的获益，而在肺鳞癌中却缺少可利用的基因突变。传统上，在治疗SQCC和ADC的策略上无明显区别。而最近越来越多的临床试验表明一些靶向药物以及新一代化疗药物对SQCC和ADC亚型患者的疗效存在很大差异。本文主要从基因组方面探讨两种肺癌之间的差异，并对SQCC中成纤维细胞生长因子受体1（fibroblast growth factor receptor 1, FGFR1）、10号染色体缺失的磷酸酶及张力蛋白同源物（gene of phosphate and tension homology deleted on chromosome ten, *PTEN*）、*PIK3CA*、血小板源性生长因子受体A（platelet-derived growth factor receptor A, PDGFRA）、盘状结构域受体2（Discoidin Domain receptor 2, DDR2）等驱动基因突变，以及针对这些基因突变的靶向治疗的进展进行综述。

早期在肺癌的化疗方案选择上只是单纯地将其分为SCLC和NSCLC，其中针对NSCLC的传统治疗策略在ADC和SQCC亚型间并无明显区别^[1]^。而最近越来越多的临床试验表明一些靶向药物以及新研发的化疗药物对ADC和SQCC亚型患者的疗效存在很大差异。如分别以EGFR和间变性淋巴瘤激酶（anaplastic lymphoma kinase, ALK）通路为靶点的分子靶向治疗EGFR酪氨酸激酶抑制剂和克唑替尼等对存在相应基因异常的ADC患者疗效明显，使部分ADC患者的生存质量和总生存期得到了明显的改善。此外，最近的一些研究表明以*BRAF*、*AKT1*、*ERBB2*基因突变和*ROS1*、*RET*融合基因为靶标的治疗对相应的ADC患者可能也是有效的。而SQCC患者往往由于缺少上述的特异性基因改变，不能从这些靶向药物获益。本文旨在从肿瘤发生、基因、表观基因、基因表达、驱动基因等方面综述SQCC的生物学特征与ADC的差异以及SQCC的潜在分子靶点的发现与研究进展。发现这两种亚型在分子水平上的差异对于研发新颖有效的治疗药物的意义重大，是推动SQCC分子靶向治疗的基础。

## 1 肺癌流行病学、肿瘤发生和生物学差异

《2012中国肿瘤登记年报》显示男性和女性发病率最高的是肺癌，死亡率最高的仍是肺癌。2014年4月14日发表的《2013中国肿瘤登记年报》显示肺癌仍居恶性肿瘤发病率及死亡率的第一位。肺癌已成为人类健康的头号杀手。尽管很多人具有或接触相同的致癌危险因素，但最终仅有一部分人发生肺癌。究其原因，肺癌的发生是一个多基因、多机制共同参与，环境因素和遗传因素共同作用的结果。

越来越多的研究^[2-6]^发现，ADC和SQCC在肿瘤生物和临床反应性方面的差异有其遗传学基础，可能与肿瘤生发的不同细胞谱系有关。绝大多数肺癌起源于支气管粘膜上皮，源于支气管腺体或肺泡上皮细胞者较少。SQCC主要起源于段和亚段支气管粘膜上皮，经鳞状上皮化生、异型增生和原位癌等阶段再演进为浸润癌；ADC来自支气管的粘膜上皮和腺上皮。由于SQCC和ADC起源于肺不同区域的不同谱系，那么这两种肿瘤发生发展所需的基因改变可能也是具有谱系限制性。换而言之，SQCC和ADC来源的细胞谱系对肺癌亚型发生过程中表现出的基因改变可能有巨大影响：①可能只有能促进肿瘤向某一种特异的恶性表型发展的基因改变才被选中并保持。②特定的基因改变和他们各自的信号传导通路的异常，可能只有在特定的细胞类型和特定的局部环境才能导致个体肺癌亚型的发生^[7]^。例如Weir等^[8]^和Bass等^[9]^分别先后在ADC和SQCC样本中发现了组织亚型特异的谱系存活癌基因（lineage-survival oncogene）*TITF1*/*NKX2*-*1*和*SOX2*的扩增。然而，仅仅是这些基因并不足以解释这两种亚型之间的差异，可能还有大量的基因与SQCC和ADC的差异化发展（differential development）相关。越来越多的研究者开始关注SQCC和ADC在分子水平上的不同，以期更好地识别与ADC和SQCC治疗密切相关的，亚型特异性的分子改变。

### 1.1 肺鳞状细胞癌整体基因组改变

在世界范围内，每年约有400, 000例患者死于SQCC。与分子靶向治疗在ADC的治疗中取得里程碑式的进展相比，SQCC尚无明确的靶向治疗药物，其生存率也较ADC低。目前，对于晚期SQCC的标准治疗仍是细胞毒性药物，大多数患者经历了一线、二线治疗后面临无药可用的状态，因此对于SQCC治疗手段的探究成了当前迫在眉睫的任务。此外，有关SQCC的基因组改变的研究也相对匮乏。早期的一些小样本研究表明SQCC和ADC在基因组不平衡、基因组改变的模式方面都有不同之处^[10]^。Sy等^[11]^分子细胞遗传学研究发现（SQCC 35例，ADC 34例）两种组织学亚型都出现+1q21-q24、+5p15-p14、+8q22-q24.1、-17p13-p12，对于比较基因组杂交数据的系统聚类模拟分析表明+2p13-p11.2、+3q25-q29、+9q13-q34、+12p、+12q12-q15、+17q21、-8p等改变与SQCC的发病机理更优先相关。两种亚型都有t（7;8）、t（8;15）、t（8;9）易位。但染色体8q和12q的易位t（8;12）仅见于ADC。Pei等^[10]^报道ADC与SQCC最明显的不同是3q24-qter的获得（SQCC *vs* ADC: 81% *vs* 35%, *P*＜0.000, 1）。相比25%的SQCC出现3q25-26扩增，只有6%的ADC出现相同的片段扩增。此外SQCC中20p13获得和4q缺失也明显多于ADCs，而6p比例过高（over representation）在ADC中较SQCC更常见。Tonon等^[12]^通过全基因组的比较基因组杂交CGH联合*ADC*与*SQCC*基因表达谱研究了18例ADC和26例SQCC样本，共发现93个灶状基因拷贝数改变，包括高幅度扩增和纯合型缺失。在这项研究中，*ADC*与*SQCC*基因组图谱基本重叠，仅有1个SQCC特异的扩增子。这个扩增子包含几个过度表达的基因，其中一个是已知调节鳞状细胞分化的基因*p63*。最近二代测序技术（next generation sequencing, NGS）的发展有助于获得大量的肿瘤基因改变的信息包括整个基因组水平上的SNP、拷贝数改变、染色体重排。2012年癌症和肿瘤基因图谱（Cancer Genome Atlas, TCGA）研究工作者对178例SQCC进行全外显子组/基因组测序、mRNA测序和mRNA表达及启动子甲基化检测，制作了SQCC的基因组全景（genomic landscape）^[4]^。这是SQCC研究的一个跨时代意义的突破。结果发现SQCC肿瘤中也存在大量复杂的基因组改变，平均每个肿瘤约有360个外显子突变，165个染色体组重排，323个基因片段拷贝数改变。在约79%的SQCC中发现了有可能做为药物靶标的基因或信号传导通路的改变，如*FGFR1*扩增和*DDR*突变等，但是SQCC的这些可以利用的改变与ADC不尽相同。因此，更好地识别SQCC与ADC肿瘤基因组的特性与差异，是推动SQCC分子靶向治疗的基础。Lockwood等^[5]^应用综合基因组学分析结合高分辨率比较基因组杂交分析和基因表达芯片图谱对来自多个相互独立队列的ADC和SQCC肿瘤样本（> 330个临床肿瘤样本）及相应的正常对照组织进行比较分析，结果发现位于染色体8p12的原癌基因BRF2（编码RNA合成酶Ⅲ转录起始因子）的过度表达出现于气道上皮细胞来源肿瘤的发展早期，仅限于SQCC，是一个新颖的谱系特异性原癌基因。BRF2通过增加合成酶Ⅲ的活性，促进细胞生长和繁殖从而导致SQCC的发展。在此基础上Lockwood等^[2]^检测和整合了261例原发NSCLC肿瘤（169例ADC和92例SQCC）的全基因组DNA拷贝数、甲基化和表达谱，进一步发现组织亚型特异的分子改变。研究者检测了两个组织亚型中基因异常频发的的非随机区域，结果发现SQCC和ADC有294个拷贝数不一致区域，其中205个是SQCC特异性，而89个是ADC特异性，而且这些拷贝数改变的状态在SQCC和ADC之间明显不同。这些组织亚型特异的拷贝数改变可能是肿瘤发生过程中优先选择的结果，决定SQCC和ADC的差异化发展和各自的组织学特征。检测位于上述亚型特异性的拷贝数改变区域内的基因表达发现968个独特基因在SQCC和ADC亚型中的表达明显不同，其中797个是SQCC特异，而171个是ADC特异的。和正常组织相比，SQCC和ADC分别有68%（447/797）和49%（71/171）的亚型特异基因在肿瘤组织中表达失调，他们很可能就是驱动肿瘤向不同亚型发展的关键基因改变^[2]^。对于上述968个基因的信号网络分析（ingenuity pathway analysis, IPA）表明SQCC中改变的基因网络主要参与DNA复制、重组和修复，以及淋巴组织结构和发展。主要的基因改变与组蛋白H4的结合与修饰，以及NFKB复合物的调节相关。相比之下，ADC中被改变的主要网络与细胞间的信号传导、发展、和药物代谢相关。SQCC和ADC基因网络改变的差异进一步表明他们不同的肿瘤生成机制。Staaf等^[3]^研究了肺癌组织及细胞株（ADC, *n*=1, 206; SQCC, *n*=467; LCC, *n*=37; SCLC, *n*=467）的基因组的全景图谱，结果发现不同的组织亚型在包括基因拷贝数改变的频率/模式、扩增、杂合性缺失（loss of heterozygosity, LOH）、肿瘤倍性和拷贝数一致的等位基因不平衡（copy-neutral allelic imbalance）等方面都不尽相同。总体来说ADC中的基因拷贝数改变和等位基因不平衡较其他亚型要少。他们进一步发现涉及染色体3q上的6个扩增和3p、4p、4q和5q上7个缺失的13个mGISTIC区域可以将ADC从SQCC中分别出来，同时SQCC中基因改变的频率总是高于ADC。关于肺癌频发基因扩增模式的研究^[3]^表明SQCC中的频发基因扩增较ADC更为频繁。

### 1.2 SQCC和ADC表观基因组图谱的差异

几个已知或推定的肿瘤抑制基因的异常甲基化频繁发生于肺癌的生成。*APC*、*p16*、*RASSF1A*（RAS association domain family 1）、*CDH13*、*RARβ*、*MGMT*、*GSTP1*是经常在肺癌中被甲基化的基因。Toyooka等^[13]^在115例NSCLC中检测上述基因的甲基化状态，发现*p16*的异常甲基化较常见于SQCC，而APC和CDH13甲基化在ADC中高于SQCC。随后，Toyooka等^[14]^收集了来自美国、澳大利亚、日本和台湾的514例NSCLC组织及84例相应的非肿瘤肺组织，进一步检测显示，ADC中*APC*、*CDH13*、和*RARβ*的甲基化率高于SQCC。近期Lockwood等^[2]^发现ADC和SQCC的总体甲基化水平无明显不同，但在DNA甲基化上两种亚型之间确实有基因特异性差异，约发现2, 384个基因的甲基化程度不同。与ADC组相比，SQCC组有明显较多的频发高甲基化和低甲基化基因位点，约占92%。

研究在ADC和SQCC亚型中分别发现了关键的致癌通路，而这些通路于这两种亚型的肿瘤生物与临床结果的差异相关。HNF4a靶基因的下调是最常见于ADC的特异通路，而在SQCC中发现大量组蛋白修饰酶以及转录因子E2F1的破坏。Heller等^[15]^在对101个NSCLC样本的全基因组CpG岛甲基化的研究中发现*HOXA2*和*HOXA10*甲基化可能与SQCC的预后相关。*HOXA2*甲基化的SQCC患者DFS明显比非*HOXA2*甲基化的SQCC患者短（中位生存期：45个月 *vs*尚未获得的数据, *P*=0.034）。同样，*HOXA10*甲基化的SQCC患者的DFS比非*HOXA10*甲基化的SQCC患者短（中位生存期：35个月vs尚未获得的数据，*P*=0.046）。但在ADC患者中没有类似的发现。

ADC和SQCC肿瘤在基因组和表观基因组水平上基因破坏的差异决定了两种NSCLC亚型的不同生物学特性。作者对49个NSCLC肿瘤的基因表达数据进行主成分分析（principal component analysis），发现778个独特的遗传学和/或表观遗传学上异常改变的基因可以清晰的将ADC和SQCC组织亚型各自聚为一类。进一步的验证试验表明同样的分析也可以将来自不同机构的两组独立样本（组1：58例ADC和53例SQCC；组2：62例ADC和76例SQCC）按照ADC和SQCC亚型精确聚类。表明这些亚型特异性遗传学和/或表观遗传学的基因改变驱动了ADC和SQCC的差异化发展。此外，上述这些ADC和SQCC亚型间遗传学、表观遗传学、基因转录水平的差异也表现在蛋白质水平，提示这些基因改变对ADC和SQCC表型有功能的影响。

### 1.3 SQCC与ADC中突变基因的差异

单一平台的研究已经发现SQCC的一些发生频率较高的基因改变，如*p63*、*PI3KCA*、*PDGFRA*、*SOX2*或*FGFR1*扩增以及*p53*、*EGFRv*Ⅲ、*PI3KCA*、*NRF2*、*PTEN*和*DDR2*等的基因突变^[16]^。2012年癌症和肿瘤基因图谱（Cancer Genome Atlas, TCGA）研究工作者对178例SQCC进行全外显子组/基因组测序、mRNA测序和mRNA表达及启动子甲基化检测制作了SQCC的基因组全景（genomic landscape）^[4]^。这是SQCC研究的一个跨时代意义的突破。分析表明96%（171/178）的肿瘤包含至少一个编码下列蛋白的基因突变：酪氨酸激酶、丝氨酸/苏氨酸激酶、PI3K催化和调节亚基、细胞核激素受体、G蛋白偶联受体、蛋白酶和酪氨酸磷酸酶。按照突变评估量表（mutation assessor score），50%到70%的突变对蛋白功能有中度到高度的影响。39%的酪氨酸激酶和42%的丝氨酸/苏氨酸激酶突变位于激酶区域，而且许多改变发生于已知的原癌基因或抑癌基因。基于美国食品药品监督管理局（Food and Drug Administration, FDA）已经认证或处于临床试验的靶向药物、RNA确认的等位基因改变以及突变评估量表等标准，64%（114/178）的SQCC患者存在潜在的靶向基因的体细胞改变。其中，3个酪氨酸激酶家族*ERBBs*、*FGFRs*和*JAKs*出现基因突变或扩增。不像ADC中*EGFR*和*KRAS*突变高发，187例SQCC样本，只有1例*KRAS*突变，2例存在对厄洛替尼和吉非替尼敏感的*EGFR*突变（Leu861Gln），但7%的SQCC有*EGFR*扩增。通过分析3个代表潜在治疗靶标的中心细胞通路PI3K/AKT、RTK和RAS的分析表明约69%的SQCC样本中有PI3K/AKT通路和RTK信号传导的改变。其中47%的肿瘤有一个PI3K/AKT通路基因的改变；26%的肿瘤有*EGFR*扩增、*BRAF*突变或*FGFR*扩增或突变导致的RTK信号通路的改变。此外PI3K/AKT通路基因改变与EGFR改变不相容。总的说来，SQCC样本中常见的基因扩增包括：*FGFR1*（15%）、*EGFR*（9%）、*PDGFRA*（9%）、*ERBB2*（4%），而基因突变包括：*FGFR2*（3%）、*BRAF*（4%）、*DDR2*（4%）、*PIK3CA*突变（16%）、*PTEN*突变/删除（15%）。2012年Paul等^[17]^运用多重PCR-直接测序法检测SQCC中已知的可以作为药物治疗靶标的驱动基因突变的发生频率包括*PIK3CA*突变、*PTEN*突变/删除、*FGFR1*扩增和*DDR*突变等，结果发现约60%的患者有可以利用的药物靶标。虽然这些新发现的基因突变或信号通路的改变在SQCC发生发展中的作用还有待于功能上的验证，这些数据为SQCC个体化治疗的研发开创了新时代。

## 2 SQCC潜在的治疗靶标和相关临床试验

### 2.1 FGFR1

FGFR属于受体酪氨酸激酶（receptor tyrosine kinase, RTKs）型受体。该受体家族主要包括FGFR1、FGFR2、FGFR3和FGFR4四个成员。FGFR主要参与调节细胞增殖、凋亡、迁移以及新生血管生成等多个过程。此外，FGFR激活突变或者配体/受体过表达导致其持续激活，也与肿瘤新生血管生成、肿瘤的侵袭与转移等过程密切相关。其中，FGFR1激活可导致通过PI3K-AKT和RAS-MEK-MAPK的下游信号，进一步影响肿瘤的发生、发展。2010年德国马克斯•普朗克神经研究所的Weiss等^[18]^检测了155例SQCC和77例其他肺肿瘤标本的高分辨率基因组图谱，发现*FGFR1*基因扩增，而且这种基因改变仅仅发生于SQCC标本。在另外一个独立的SQCC患者群体中进一步验证约有22%的SQCC标本有*FGFR1*基因扩增。作者进一步通过高通量的细胞筛选技术，分析FGFR1抑制剂（PD173074）对83例肺癌细胞株生长的影响。结果发现FGFR1抑制剂特异性地只对那些含有*FGFR1*基因扩增的细胞表现出明显的抑制细胞生长和诱导细胞凋亡的作用。Kim等^[19]^发现262例韩国SQCC患者中34（13%）例*FGFR1*扩增阳性（*FGFR1* amp+，*FGFR1*基因拷贝数≥9），而且*FGFR1* amp+可以作为SQCC患者预后差的指标。*FGFR1* amp+ SQCC患者的无疾病生存期26.9个月 *vs* 94.6个月，*P*＜0.000, 1）和总生存期（OS：51.2个月 *vs* 115.0个月，*P*=0.002）都较无扩增的患者明显缩短。此外，*FGFR1* amp+发生率与吸烟状态相关（吸烟*vs*戒烟*vs*非吸烟：28.9% *vs* 2.5% *vs* 0%; *P*=0.001），表明*FGFR1* amp+可能是一种由吸烟导致的原癌基因改变。Heist等^[20]^对于226例白种SQCC患者的研究发现16%患者为*FGFR1* amp+，而*FGFR1*扩增状态与患者临床分期、生存期、吸烟史等无关。这两个研究结果的差异可能与人种及*FGFR1*扩增的定义的差异有关。综上所述，FGFR1是SQCC中一个常见的基因改变，与肿瘤细胞的生长和存活密切相关，是一个潜在的SQCC的药物治疗靶标。FGFR1抑制剂一类的药物可能对治疗*FGFR1* amp+的SQCC有效。

### 2.2 磷脂酰肌醇3-激酶（phosphatidylinositol 3-kinase, PI3K）通路

PI3K是一种脂激酶，该脂激酶由调节亚单位p85和催化亚单位p110组成。PI3K通过催化PIP2磷酸化为PIP3进一步激活下游的Akt，与多种底物结合后，调节细胞的增殖、分化、凋亡以及迁移。大量的遗传和实验室研究表明PI3K-AKt通路对肿瘤细胞的生长和存活至关重要。1994年Volinia^[21]^利用原位杂交技术检测到*PIK3CA*基因，*PIK3CA*突变约4/5发生在螺旋区（外显子9）和激酶区（外显子20）这两个热点区域。其突变不仅可以减少细胞的凋亡还可以促进肿瘤的浸润、提高其下游激酶PI3K的活性。PI3K信号通路参与了体内多种肿瘤的发生（如肺癌、肠癌、乳腺癌、前列腺癌等），对于NSCLC而言，PIK3CA（编码p110α催化亚基）的突变或异常扩增在鳞癌和非鳞癌略有不同，SQCC略高于ADC^[22-24]^。据报道^[4, 17, 22]^SQCC中*PIK3CA*基因突变和扩增的发生率分别为8%-16%和33%-43%。目前，PI3K/AKT/mTOR抑制剂[包括CAL-101（IC87114）、NVP-BEZ235、BKM-120、GDC-0941、ZSTK474等]尚在临床试验阶段。

肿瘤抑癌基因*PTEN*却与PI3K的功能相反。*PTEN*基因是1997年被发现的，位于染色体10q23.3，由9个外显子和8个内含子组成，编码403个氨基酸组成的蛋白质。该抑癌基因同时具有脂质磷酸酶和蛋白磷酸酶双重活性。PTEN能使PIP3去磷酸化为PIP2，对PI3K-AKT信号起到一个负调控的作用。因此突变的PTEN，尤其是起负调控作用，进一步导致肿瘤进行性生长。PTEN主要是通过等位基因的缺失、基因突变和甲基化方式使其失活而失去了对细胞周期和细胞生长的负调控、促进细胞凋亡和抑制肿瘤的发生。*PTEN*的突变/缺失见于15%-28%的SQCC^[4, 17, 25]^。有证据^[26]^显示，PTEN蛋白的表达及*PTEN*基因的突变情况与多种恶性肿瘤有关。多项研究^[27-30]^表明，在NSCLC中，PTEN蛋白表达水平与肺癌的恶性程度成反比，蛋白表达水平越低，肺癌恶性程度越高。并且，无淋巴结转移组PTEN阳性表达率高于有淋巴结转移组，这说明PTEN失表达的患者更容易发生淋巴结转移。同时，还检测出，ADC中PTEN蛋白表达明显高于SQCC，表明PTEN蛋白的丢失在SQCC中的作用可能高于ADC。抑癌基因*PTEN*在肺癌的发生、发展中起重要作用，与肺癌的发生、细胞增殖、浸润和转移关系密切。因此，抑癌基因*PTEN*有可能成为判断SQCC恶性程度、转移和预后的指标之一。针对PI3K/AKT/mTOR信号传导通路的靶向药物对于PTEN缺失的肿瘤可能是有效的。值得一提的是PIK3CB在PTEN缺失的肿瘤中扮演至关重要的角色。在一个前列腺癌模型中，抑制PIK3CB降低AKT的磷酸化和阻止肿瘤生成^[31]^。另一项研究^[32]^揭示，在三个PTEN缺失的肿瘤细胞株中PIK3CA耗竭并不影响PI3K信号传导或细胞生长；相反，PI3K信号传导的抑制依赖于PIK3CB的下调。综上所述，选择性的抑制PIK3CB在治疗PTEN缺失的肿瘤中可能是有效的。

### 2.3 PDGFRA

PDGF是从人的血小板中分离出来的促血管生成因子，同时它也是成纤维细胞、平滑肌细胞以及其他间充质来源细胞的强有丝分裂原和化学驱动剂。PDGFR是酪氨酸蛋白激酶家族成员（包括PDGFRA和PDGFRB），能够促进细胞的趋化、分裂与增殖；在机体生长发育、创伤修复等生理过程中起着积极重要的作用^[33, 34]^。血小板源性生长因子及其受体过度激活和异常表达可诱导肿瘤新生血管的形成，直接或间接地促进肿瘤细胞增殖与迁移。还可以降解细胞外基质，使癌细胞表面粘附分子减少，直接或间接参与肿瘤的侵袭与转移。文献^[4, 35]^报道，PDGFR-α（位于染色体4q12）扩增约见于9%的SQCC样本。目前已有多个多靶点的TKIs以PDGFRA为靶向，如索拉非尼、舒尼替尼以及伊马替尼。虽然抗PDGFRA似乎是一种有潜力的治疗策略，但目前的研究结果不尽人意。在一项Ⅲ期随机临床试验中在以铂类为基础的化疗中加入舒尼替尼并不能改善SQCC的生存期。更有甚者，添加索拉非尼增加SQCC患者的死亡率^[36]^。在脑胶质瘤和胃肠间质瘤的Ⅱ期临床试验中^[16]^一个选择性crenolanib的抗肿瘤疗效正在被测试（NCT01229644, NCT01243346）。舒尼替尼是被批准的在临床上使用的多靶点的酪氨酸激酶抑制剂。它主要抑制血管内皮生长因子受体（VEGF receptor, VEGFR）、c-KIT和PDGFR。McDermott等^[37]^在NCIH1703腺鳞癌的NSCLC细胞系中，发现在肺鳞状细胞癌中有PDGFRA的扩增。通过shRNA敲除或小分子抑制PDGFRA可损害细胞存活，表明PDGFRA可能是含*PDGFRA*基因扩增的癌症的一种新的驱动癌基因。除舒尼替尼外，多种PDGFR抑制剂目前在临床开发中。通过对PDGFRA和其相应抑制剂的研究，将会为SQCC的治疗提供新的思路。

### 2.4 DDR2

DDR2是一种酪氨酸激酶受体，配体胶原蛋白与受体相结合导致受体磷酸化，进而促进细胞迁移、增殖和存活^[38]^。2011年，Hammerman等^[39]^检测290个SQCC患者标本发现约3.8%的患者存在*DDR2*的基因突变。为了进一步评估是否针对*DDR2*的基因突变治疗可以成为对SQCC的新的治疗策略。Williams^[34]^进一步发现用RNAi敲除DDR2，或用多靶点的酪氨酸酶抑制剂达沙替尼都可以选择性地杀死存在*DDR2*突变的SQCC细胞株。在细胞中表达突变的*DDR2*基因可以导致细胞恶性转化，而达沙替尼可以抑制这种转化。此外，在异体移植型肿瘤的动物模型中，达沙替尼可以有效抑制存在*DDR2*突变的人类SQCC细胞株接种裸鼠产生的肿瘤。由此可见，*DDR2*突变在SQCC中可能是一个有前景的治疗靶标。达沙替尼被FDA批准，主要用于治疗BCR-ABL的慢性髓细胞白血病、急性淋巴细胞白血病、c-KIT的胃肠道间质瘤、以及PDGFR的慢性粒-单核细胞白血病。目前一个验证达沙替尼对*DDR2*突变的SQCC患者疗效的Ⅱ期临床试验正在进行（NCT01491633）^[16]^。SQCC的*DDR2*突变率虽然不高，与ADC中*EML4*-*ALK*融合基因的发生率相近，但若经进一步临床研究证实达沙替尼对*DDR2*突变的SQCC患者有效，并且针对该药的毒副作用相应的进行处理，该亚型患者的治疗将发生革命性进展。

SQCC患者的治疗手段仍较局限，我们亟需寻找新的方法。美国礼来公司^[40]^正在进行一项关于NSCLC中的SQCC的Ⅲ期随机多中心的开放性临床实验。该实验主要是通过吉西他滨-顺铂和necitumumab（GC+N）联合用于治疗Ⅳ期SQCC的患者与吉西他滨和顺铂标准治疗进行对比。这是迄今为止，关于NSCLC中的SQCC最大的一项Ⅲ期临床实验。该实验共招募了1, 093例SQCC患者，釆取随机分组双盲比较设计，在全球多中心展开。主要临床终点为总存活时间（overall survival, OS），次要临床终点为无进展存活时间（progression free survival, PFS）和总应答率（overall response rate, ORR）。在今年的ASCO会议上，美国礼来公司公布了该实验的阳性结果，结果显示，necitumumab加化疗与对照组（化疗本身）相比可以明显延长SQCC患者的总体存活时间；OS分别为11.5个月和9.9个月（HR=0.84, *P*=0.012）。抗体联合用药的副作用在可以耐受的范围。Necitumumab是历史上第一个证明可以延长SQCC患者生存率的生物药物。

## 3 小结与展望

目前晚期SQCC的治疗手段较为局限，含铂双药联合化疗仍然为其标准治疗方案，可提供给医师及患者调整的治疗方案的有效药物很少，现有的肺癌相关分子靶向治疗药物在SQCC面前显得束手无策，迫切需要对SQCC的治疗进行更深入的探究。纵观目前的临床研究，诸如FGFR1、DDR2等治疗靶点已初露端倪，并且如nab-PC等多种已上市药物在SQCC方面的治疗优势也被渐渐发掘出来，虽然这些药物的治疗效果仍需大规模的前瞻性临床研究去证实，但这些临床前的数据已足以让我们为之振奋。综上所述，SQCC的治疗正在进行一场变革，在这场变革中分子靶向治疗占据着举足轻重的地位，对肺癌的研究重点也将逐渐从ADC转移向SQCC。
